# Integrative Analysis Toward Different Glucose Tolerance-Related Gut Microbiota and Diet

**DOI:** 10.3389/fendo.2019.00295

**Published:** 2019-05-27

**Authors:** Rebiya Nuli, Junxiu Cai, Aizhatiguli Kadeer, Yangyi Zhang, Patamu Mohemaiti

**Affiliations:** ^1^School of Public Health, Xinjiang Medical University, Ürümqi, China; ^2^College of Basic Medical Science, Xinjiang Medical University, Ürümqi, China; ^3^Medical Department, The Fifth Affiliated Hospital of Xinjiang Medical University, Ürümqi, China; ^4^Health Management Institute, The First Affiliated Hospital of Xinjiang Medical University, Ürümqi, China

**Keywords:** gut microbiota, 16S rRNA, impaired glucose regulation, type 2 diabetes, Uyghur, dietary survey

## Abstract

**Objective:** There is evidence that type 2 diabetes (T2DM) is affected by gut microbiota, and gut microbiota diversity modified by diet. To investigate its modifications in Uyghur patients with different glucose tolerance, we enrolled 561 subjects: newly diagnosed T2DM (*n* = 145), impaired glucose regulation (IGR) patients (*n* = 138) and in normal control (NC) population (*n* = 278).

**Methods:** The nutrient intake in food frequency questionnaire was calculated by R language. The regions V3-V4 of 16S ribosomal RNA were sequenced by using Illumina Miseq platform. Sequences were clustered by operational taxonomy units, gut microbiota composition, and diversity was analyzed. Correlations between bacterial composition at different level and dietary factors were evaluated.

**Results:** The α-diversity was highest in NC, followed by T2DM and IGR; β-diversity distinguished between patients and NC. Compared to NC, *Saccharibacteria* was significantly increased in T2DM and IGR. *Deferribacteres* was significantly increased in T2DM compared to NC and IGR. *Veillonella, Pasteurellaceae*, and *Haemophilus* were over-represented in IGR. Abundance of *Bacteroidetes* was negatively correlated with LDL-C; Abundance of *Tenericutes* was negatively correlated with hip circumference and total cholesterol, positively correlated with HDL-C and cake intake; *Actinobacteria* was positively correlated with BMI and folic acid intake, negatively correlated with oil intake. *Firmicutes* was negatively correlated with beverage and alcohol intake. *Spirochaetae* was negatively correlated with fungus, fruits, beans, vitamin C, dietary fiber, and calcium. *Fusobacteria* was positively correlated with beans intake, and was negatively correlated with fat intake. *Proteobacteria* was positively correlated with tuber crops intake. *Synergistetes* was positively correlated with cholesterol, nicotinic acid, and selenium intake. *Deferribacteres* was negatively correlated with magnesium intake.

**Conclusions:** At the phylum and genus level, the structure and diversity of intestinal microbiota of T2DM and IGR was altered, the number of OTUs, the relative abundance, and diversity were all decreased. The gut microbiota of the newly diagnosed T2DM, IGR, and NC were related to age, blood lipids, BMI, blood pressure, and dietary nutrient intake. Unbalanced nutrient intake in the three groups may affect the structure and abundance of the gut microbiota, which may play a role in the occurrence and development of T2DM.

## Introduction

Recent studies on Type 2 diabetes mellitus (T2DM) reported that there are direct links between diet, gut microbiota, and biological metabolic markers (1). Gut microbiota is associated with metabolic diseases such as obesity and diabetes, and the changes in the gut microbiota diversity are one of the important environmental risk factors for metabolic disease (2). In addition, gut microbiota is also with diverse body functions like dietary calorific bioavailability (3). T2DM and obesity are characterized by insulin resistance and low levels of inflammatory response, while the gut microbiota regulates host energy balance and inflammatory response and is closely related to the occurrence of metabolic diseases (4). The gut microbiota is rich in diversity in normal people, while the reduced microbial diversity and dysbiosis are linked with obesity, T2DM, and low inflammation (5). According to the forecast of international diabetes federation (IDF) in 2015, the number of T2DM patients in China will increase to 150 million by 2040, and the increase in T2DM patients is proportional to the increase in obesity. The incidence of diabetes (including type 1 and type 2) in China has increased from 0.9% in 1980 to 11.6% in 2013. The number of diabetic patients in the country has reached 100 million, and it is increasing year by year, and another 50.1% of adults are in the pre-diabetes status (6). Diabetes is top risk factor for health in Xinjiang of China, and the incidence rate in the population is as high as 10.47% (7). Diabetes is a chronic disease, and impaired glucose regulation (IGR) is a pre-diabetic status of diabetes, including impaired fasting glucose (IFG), and impaired glucose tolerance (IGT). According to IDF statistics, as of 2015, 352 million of the world's population are IGR patients, with a prevalence rate of 14.1%. In recent years, the number of IGR patients in China has increasing, and the prevalence rate has increased from 15.5% in 2008 to 35.7% in 2013 (8). Pre-diabetes can progress to diabetes or cardiovascular disease (9), and about 9–20.8% of IGR patients develop diabetes within 3 years (10).

Diet is an important factor in regulating gut microbiota. Regulating gut microbiota imbalance through dietary pathways has become a new research direction. The investigation and analysis of dietary structure and dietary adjustment have also played an increasingly important role in the study of gut microbiota and metabolic diseases (11). By integrating dietary and metagenomics information, we would be better understanding the interplay between diet, gut microbiota, and host metabolism.

To explore potential characteristics of gut microbiota that associated with newly diagnosed T2DM and IGR sufferer, dietary survey and gut microbial diversity analysis are applied to find relationship between specific dietary pattern and gut microbiota diversity.

## Materials and Methods

A total of 561 Uyghur subjects aged 30–70 years old were enrolled in the present study from six community health service centers of Urumqi, Xinjiang Uyghur Autonomous Region of China. Among them, 145 subjects were newly diagnosed with T2DM by oral glucose tolerance test (OGTT). They did not use any hypoglycemic drugs by that time. One hundred and thirty eight subjects were grouped in IGR. We defined diabetes by using the American Diabetes Association (ADA) 2014 criteria. The NC group comprised 278 normoglycemic subjects who were randomly selected and matched for age, gender to cases from the general population. We excluded those subjects who reported already having diabetes, cardiovascular disease (myocardial infarction, angina pectoris, coronary artery surgery, or stroke), kidney disease and cancer because diagnosis of these diseases may affect diet. Pregnant women, lactating women were not included in the study. People who could not provide written consent for the study, or who had neurological impairments, and/or severe mental illness were excluded from the study.

The study protocol was approved by the Ethics Committee of the firth affiliated Hospital of Xinjiang Medical University. Written informed consent was obtained from all subjects.

### Assessments of Anthropometric and Biochemical Measurements

Anthropometric measurements data, including gender, age were collected. Height, weight, waist, and hip circumference (WC, HC) were measured. Body mass index (BMI) was calculated. Fasting plasma glucose (FPG), 2 h OGTT, total cholesterol (TC), triglyceride (TG), high-density lipoprotein cholesterol (HDL-C), low-density lipoprotein cholesterol (LDL-C) were measured by chemical analysis (Beckman Coulter AU5800 Clinical Chemistry System, Newark, USA).

### Dietary Assessment

Food consumption including the type and amount of food and meal was collected by using a validated semi-quantitative food frequency questionnaire (SQFFQ), referring to nutrition, and health status survey of Chinese residents of 2010 and based on the local Chinese Uyghur population diet characteristics in Urumqi of Xinjiang. The SQFFQ included 84 food items and 16 categories, which covered 90% of the commonly consumed foods of Chinese Uyghur population in Urumqi. All participants were asked to recall the frequency of each food item over the previous 12 months and the estimated portion size by making comparisons with the specified reference portion. Participants were requested to recall the frequency of consumption of each food item (daily, weekly, monthly, annually, never). The amount of vegetable oil, animal oil, and salt were asked according to the family consumption per month as the unit. Data was converted into grams (g)/day. The daily food intake, caloric intake and various nutrients are calculated by converting into an adult male (that is, a standard person) according to age and sex in the following analysis. The Chinese Food Composition Tables (Yang YX, Wang GY, Pan XC. China Food Composition. Beijing: Beijing University Medical Press, 2009) were used to estimate intake of dietary energy (kcal/day), macronutrients and micronutrients.

The SQFFQ was designed for and validated in this population. Pearson correlation coefficients for reliability of the SQFFQ ranged from 0.65 to 0.91. The average correlation coefficient is 0.73. Generally, these data indicate that the SQFFQ provides reasonably valid and reliable measures of the average long-term dietary intake.

### Microbial Diversity Analysis

Stool samples were collected from 20 newly diagnosed T2DM patients, 20 IGR patients, and 20 NC who were selected by inclusion criteria. Microbial DNA was extracted from stool sample using the QIAamp DNA Stool Mini Kit (Qiagen, Germany) according to manufacturer's protocols. The V3-V4 region of the 16S ribosomal RNA was amplified for sequencing using Illumina Miseq platform (Illumina, San Diego, USA) according to the standard protocols by Major Bio-Pharm Technology Co. Ltd., (Shanghai, China). Raw fastq files were demultiplexed, quality-filtered by Trimmomatic and merged by FLASH with the following criteria: (i) the reads were truncated at any site receiving an average quality score <20 over a 50 bp sliding window. (ii) Primers were exactly matched allowing 2 nucleotide mismatching, and reads containing ambiguous bases were removed. (iii) Sequences whose overlap longer than 10 bp were merged according to their overlap sequence. Operational taxonomic units (OTUs) were clustered with 97% similarity cutoff using PARSE (version 7.1 http://drive5.com/uparse/) and chimeric sequences were identified and removed using UCHIME. The taxonomy of each 16S rRNA gene sequence was analyzed by RDP Classifier algorithm (http://rdp.cme.msu.edu/) against the Silva (SSU123) 16S rRNA database using confidence threshold of 70%.

### Univariate Statistical Analysis

All the analyses were performed by using SPSS version 21. The Shapiro-Wilk test, Q-Q plots, and histograms were applied to ensure the normal distribution of variables. Log transformation was performed for non-normally distributed variables.

Sample characteristics were presented as mean values and standard deviations for continuous variables, and percentages for categorical variables. Baseline characteristics were compared between three groups by using the analysis of chi-square test (categorical variables) and student's *T*-test or one-way ANOVA test (continuous variables). A Kruskal-Wallis H rank non-parametric test was used for non-normally distributed variables.

The nutrient intakes of each sample in semi quantitative frequency questionnaire were calculated by R language (version 3.2.2). Differences in α-diversity were tested using student's *T*-test or one-way ANOVA test. Differences in microbiota composition as assessed by β-diversity metrics were tested by one-way ANOVA.

### Multivariate Statistical Analysis

Correlations between gut microbiota and dietary intake were analyzed by RDA/CCA test implemented in R vegan package and p heatmap package. Linear discriminant effect size analysis (LEfSe) based on the non-parametric factorial Kruskal-Wallis test was performed using the default parameters at phylum to genus taxonomic level to find microbial biomarkers for the gut microbiota of T2DM and IGR groups. LEfSe used linear discriminant analysis (LDA) to estimate the effect of the abundance of each component (species) on the difference effect. The threshold on the linear discriminant analysis (LDA) score for discriminative biomarkers was 2.0. All statistical analyses were conducted using R 3.2.2.

## Results

A total of 561 participants (270 females, 291 males) were included in this study. The characteristics of the study population were shown in Table 1. The average values of age, WC, SBP, FPG, and TC of the T2DM and IGR patients were significantly (*P* < 0.01) higher than that of the NC, and the HC, DBP of the NC were significantly lower than that of T2DM group (*P* < 0.01). TG and LDL-C in the NC group were significantly lower than in T2DM and IGR groups. There were not statistically significant among three groups by gender, smoking status, and HDL-C.

**Table 1:** Clinical characteristics of three groups.

**Parameters**	**T2DM group (*n* = 145)**	**IGR group (*n* = 138)**	**NC group (*n* = 278)**	**χ^**2**^/F**	***P***
Sex (male/female)	75/69	65/73	129/149	1.46	>0.05
Age (years)	53.31 ± 9.84	48.87 ± 10.62	42.95 ± 8.65	60.2	<0.01
BMI (kg/m^2^)	28.81 ± 4.06	28.31 ± 4.54	26.82 ± 4.35	11.95	<0.01
WC (cm)	102.97 ± 10.03	97.21 ± 12.31	92.08 ± 12.30	41.63	<0.01
HC (cm)	107.55 ± 10.03	105.40 ± 10.10	102.45 ± 9.87	13.17	<0.01
SBP (mmHg)	134.04 ± 17.49	128.44 ± 20.32	119.93 ± 16.27	32.73	<0.01
DBP (mmHg)	80.59 ± 10.00	79.53 ± 12.84	74.44 ± 11.73	16.85	<0.01
FPG (mmol/L)	9.50 ± 3.91	6.23 ± 0.43	5.06 ± 0.40	233.24	<0.01
TC (mmol/L)	4.85 ± 1.39	4.71 ± 1.12	4.20 ± 0.93	20.27	<0.01
TG (mmol/L)	2.84 ± 2.74	2.06 ± 1.25	1.78 ± 1.23	17.37	<0.01
LDL-C (mmol/L)	2.76 ± 0.92	2.64 ± 0.93	2.23 ± 0.70	23.73	<0.01
HDL-C (mmol/L)	1.54 ± 1.05	1.61 ± 1.56	1.57 ± 0.54	0.2	>0.05
Smoking status (smoke/not smoke)	28/117	31/107	66/212	1.084	>0.05

*BMI, body mass index; WC, waist circumference; HC, hip circumference; SBP, systolic blood pressure; DBP, diastolic blood pressure; FPG, Fasting plasma glucose; TC, total cholesterol; TG, triglyceride; LDL-C, low-density lipoprotein cholesterol; HDL-C, high-density lipoprotein cholesterol*.

### Dietary Intake Analysis

Compared with the recommended amount of Chinese dietary guidelines, the intakes of vegetables, fish, shrimp and dairy products were insufficient for T2DM patients, and the intakes of cereals, meat, salt, and oil were excessive (Tables 2, 3).

**Table 2:** Daily average intake of staple food and soybean nuts in T2DM group.

**Foods**	**Cereals**	**Tuber crop**	**Cereal potato and miscellaneous grains**	**Beans**	**Nuts**	**Beans and nuts**
Daily intake(g)	477.89	64.54	542.43	21.35	67.25	88.60
Recommended amount (g)	250~400			30~50		
Ratio (%)	135.61			177.20		

**Table 3:** Daily average intake of other foods in T2DM group.

**Foods**	**Vegetables**	**Fruits**	**Fish, Shrimp**	**Dairy products**	**Egg**
Daily intake(g)	279.09	**354.50**	10.23	209.55	43.13
Recommended amount(g)	300~500	200~400	50~100	300	25~30
Ratio (%)	93.03	163.50	20.46	69.85	143.77
**Foods**	**Meats**	**Water**	**Salt**	**Oil**	
Daily intake(g)	151.55	**1481.80**	6.75	38.67	
Recommended amount(g)	50~70	1200	6	25~30	
Ratio (%)	216.50	123.48	112.50	128.90	

*Bold values indicate compared with recommended nutrient intakes (RNI), the intakes of calcium, and iodine were insufficient in T2DM patients*.

Compared with recommended nutrient intakes (RNI), the intakes of vitamin B6, vitamin D, folic acid, calcium, and iodine were insufficient in T2DM patients, and intakes of fat, nicotinic acid, vitamin E, potassium, iron, copper, and manganese were excessive (Tables 4, 5).

**Table 4:** Comparison of daily vitamin intake and recommended amount in T2DM group.

**Vitamin**	**Daily intake**	**RNI**	**Ratio (%)**
Vitamin A (μgRE)	1053.08	800	131.64
Vitamin B1 (mg)	1.48	1.4	105.71
Vitamin B2 (mg)	1.50	1.4	107.14
Vitamin B6 (mg)	0.47	1.4	**33.57**
Vitamin C (mg)	131.10	100	131.10
Vitamin D (μg)	1.51	10	**15.10**
Vitamin E (mg)	35.20	14	251.43
Folic acid (μg)	113.16	400	**28.29**
Nicotinic acid (mg)	22.40	14	160.00

*Bold values indicate compared with recommended nutrient intakes (RNI), the intakes of calcium, and iodine were insufficient in T2DM patients*.

**Table 5:** Comparison of daily mineral intake and recommendation amount in T2DM group.

**Minerals**	**Daily intake**	**RNI**	**Ratio (%)**
Calcium (mg)	642.93	800	**80.37**
Phosphorus (mg)	971.82	720	134.98
Potassium (mg)	2515.91	2000	125.80
Sodium (mg)	3026.47	2200	137.57
Magnesium (mg)	410.51	330	124.40
Iron (mg)	28.18	20	140.90
Iodine (μg)	32.49	150	**21.66**
Zinc (mg)	17.22	12.5	137.76
Selenium (μg)	78.95	60	131.58
Copper (mg)	4.00	2	200.00
Manganese (mg)	7.14	4.5	158.67

*Bold values indicate compared with recommended nutrient intakes (RNI), the intakes of calcium, and iodine were insufficient in T2DM patients*.

The daily intakes of vegetables, tuber crop, fruits, nuts, cakes, cholesterol, vitamin B6, vitamin E, folic acid, calcium, phosphorus and sodium were statistically different in three groups (*P* < 0.05) (Table S1).

### Gut Microbiota Diversity

#### Sequencing Coverage and Bacterial Diversity Analysis

The participants' clinical information was listed in Table 6. We obtained 3,269,951 usable optimized raw sequences; average length of optimized sequence is 436.82 bp. The sequences were clustered into OTU. Seven hundred and twenty six OTUs were clustered form 60 samples by bioinformatics statistical analysis, and species classification information of each OTU was obtained.

**Table 6:** Comparison of clinical characteristics between three groups.

**Parameters**	**T2DM (*n* = 20)**	**IGR (*n* = 20)**	**NGT (*n* = 20)**	**χ^**2**^/F**	***P***
Sex (male/female)	11/9	12/8	12/8	0.14	0.934
Age (years)	49.85 ± 11.40	45.55 ± 7.14	45.30 ± 3.47	2.04	0.140
BMI (Kg/m^2^)	25.76 ± 2.03	24.42 ± 4.24	23.83 ± 3.03	1.87	0.164
SBP (mmHg)	131.70 ± 21.74	129.70 ± 16.56	120.30 ± 16.55	2.18	0.123
DBP (mmHg)	78.10 ± 10.06	80.75 ± 10.10	73.20 ± 12.64	2.43	0.098
FPG (mmol/L)	8.58 ± 2.23	6.49 ± 0.28	4.87 ± 0.29	40.60	<0.001
TC (mmol/L)	4.31 ± 0.92	4.42 ± 0.78	4.02 ± 1.09	0.96	0.388
TG (mmol/L)	2.10 ± 0.84	2.21 ± 1.09	1.87 ± 1.48	0.44	0.645
LDL-C (mmol/L)	2.31 ± 0.51	2.41 ± 0.68	2.31 ± 0.72	0.16	0.856
HDL-C (mmol/L)	1.60 ± 0.0.36	1.77 ± 0.73	1.55 ± 0.63	0.82	0.448
Smoking status (smoke/not smoke)	5/15	7/13	10/10	2.73	0.256

*BMI, body mass index; WC, waist circumference; HC, hip circumference; SBP, systolic blood pressure; DBP, diastolic blood pressure; FPG, Fasting plasma glucose; TC, total cholesterol; TG, triglyceride; LDL-C, low-density lipoprotein cholesterol; HDL-C, high-density lipoprotein cholesterol*.

**Figure 1:**
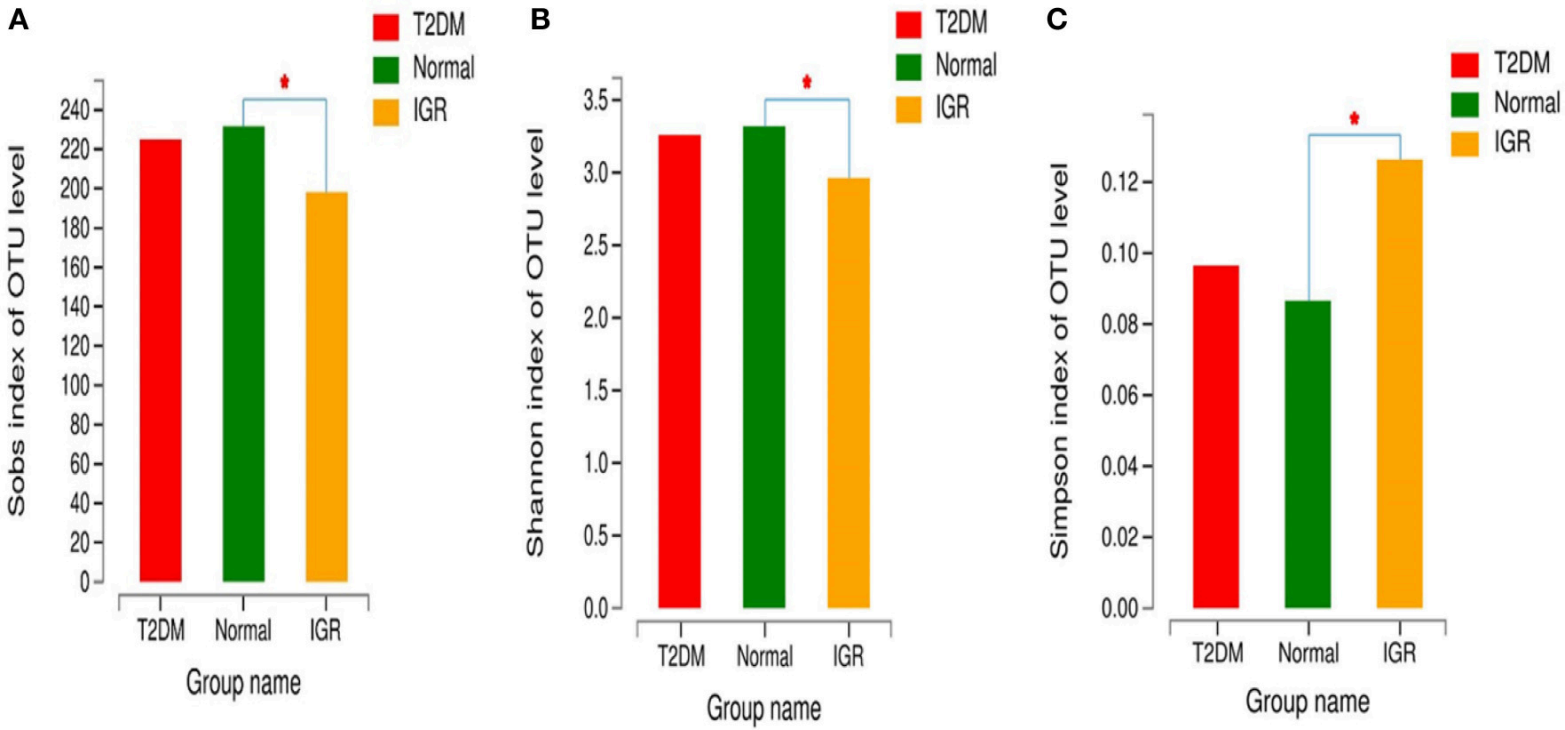
Comparison of α-diversity indexes in T2DM, IGR, and NC groups. ^*^Compare two group, *P* < 0.05 **(A)** α diversity-Sobs index bar chart **(B)** α diversity-Shannon index bar chart **(C)** α diversity-Simpson index bar chart.

Changes in the richness and diversity of the gut microbiota were estimated by Sobs index, Shannon index, Simpson index, ACE index, and Chao index. The differences of Shannon index, Sobs index, and Simpson index were statistically significant in three groups (*P* < 0.05; Table 7). Sobs index and Shannon index of IGR were significantly lower than that of NC, Simpson index of IGR was significantly higher than that of NC (*P* < 0.05). No significant differences were detected between T2DM and NC groups, T2DM and IGR based on indexes reflecting the α-diversity (Figure 1).

**Table 7:** Comparison of microbiota diversity index between three groups.

**Diversity index**	**T2DM group (*n* = 20)**	**IGR group (*n* = 20)**	**NC group (*n* = 20)**
Sobs index	225.1 ± 66.80	198.25 ± 52.03^*^	231.7 ± 45.31
Shannon index	3.26 ± 0.50	2.96 ± 0.56^*^	3.32 ± 0.47
Simpson index	0.1 ± 0.06	0.13 ± 0.08^*^	0.087 ± 0.04
ACE index	266.66 ± 64.43	244.6 ± 46.63	263 ± 49.96
Chao index	267.74 ± 54.26	244.94 ± 54.26	266.66 ± 47.99

* *IGR vs. NC, P ≤ 0.05*.

### Gut Microbiota at Different Taxonomic Levels

**Figure 2:**
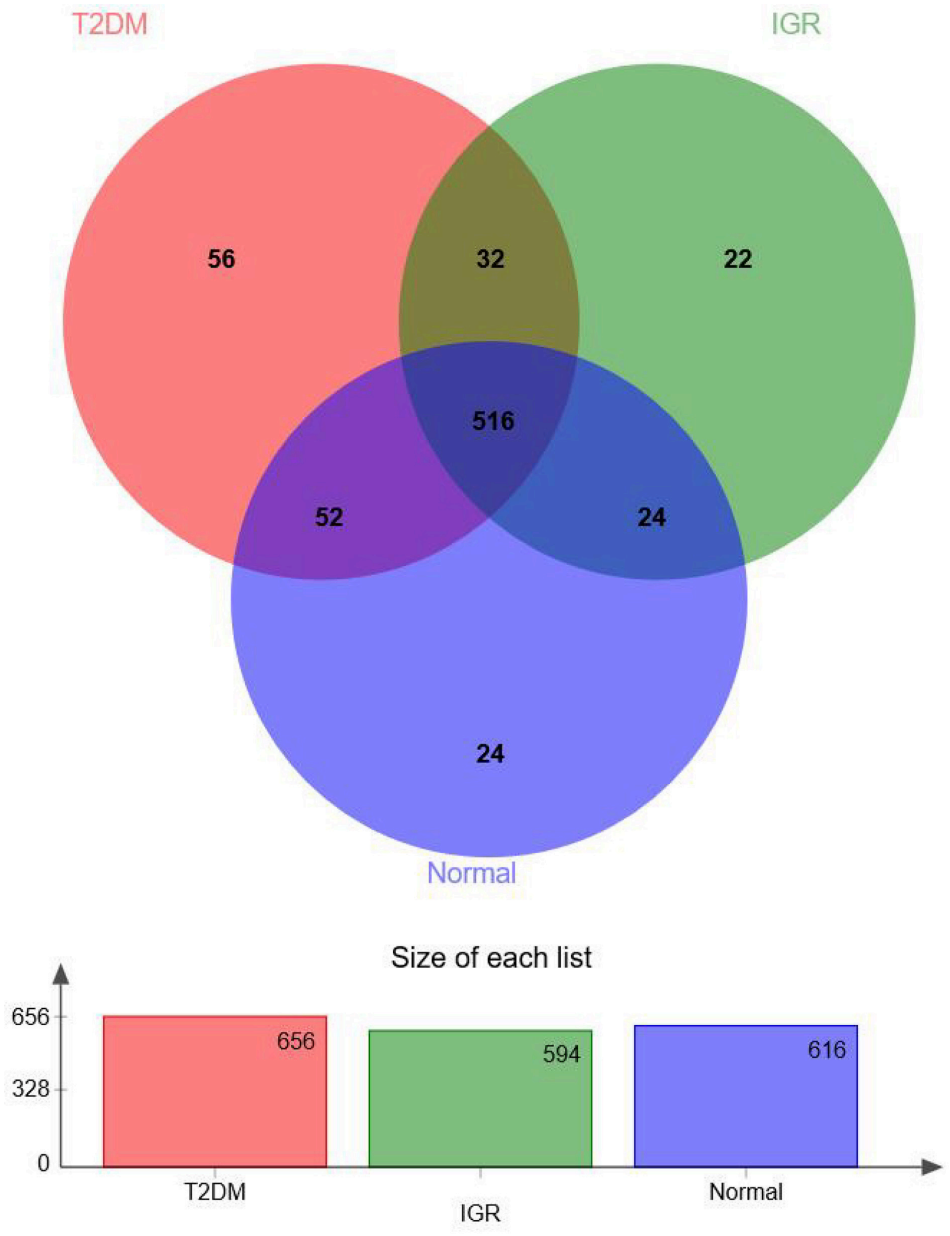
Venn map of OTUs.

According to the Venn map analysis, it was found that there were 516 common OTU of the three groups, 540 species were common in IGR and NC, 548 species were common in the IGR, and T2DM, and 568 species were common in the T2DM and NC. There were 22 species specific to the IGR group, 56 species specific to the T2DM group, and 24 species specific in the normal group (Figure 2).

**Figure 3:**
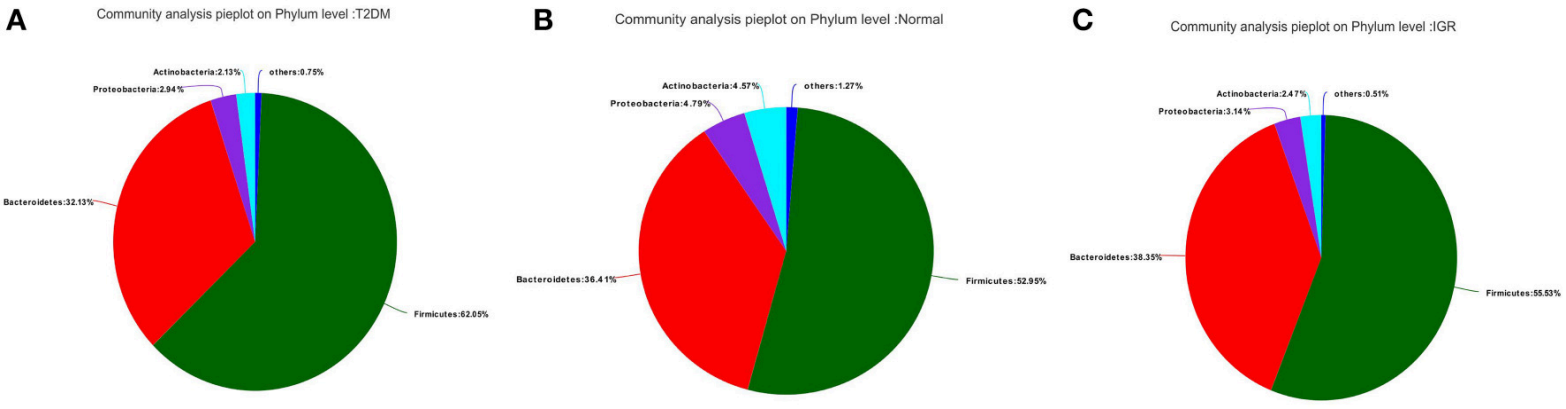
Pie charts of gut microbiota at phylum level in three groups. **(A)** T2DM group **(B)** NC group **(C)** IGR group.

**Figure 4:**
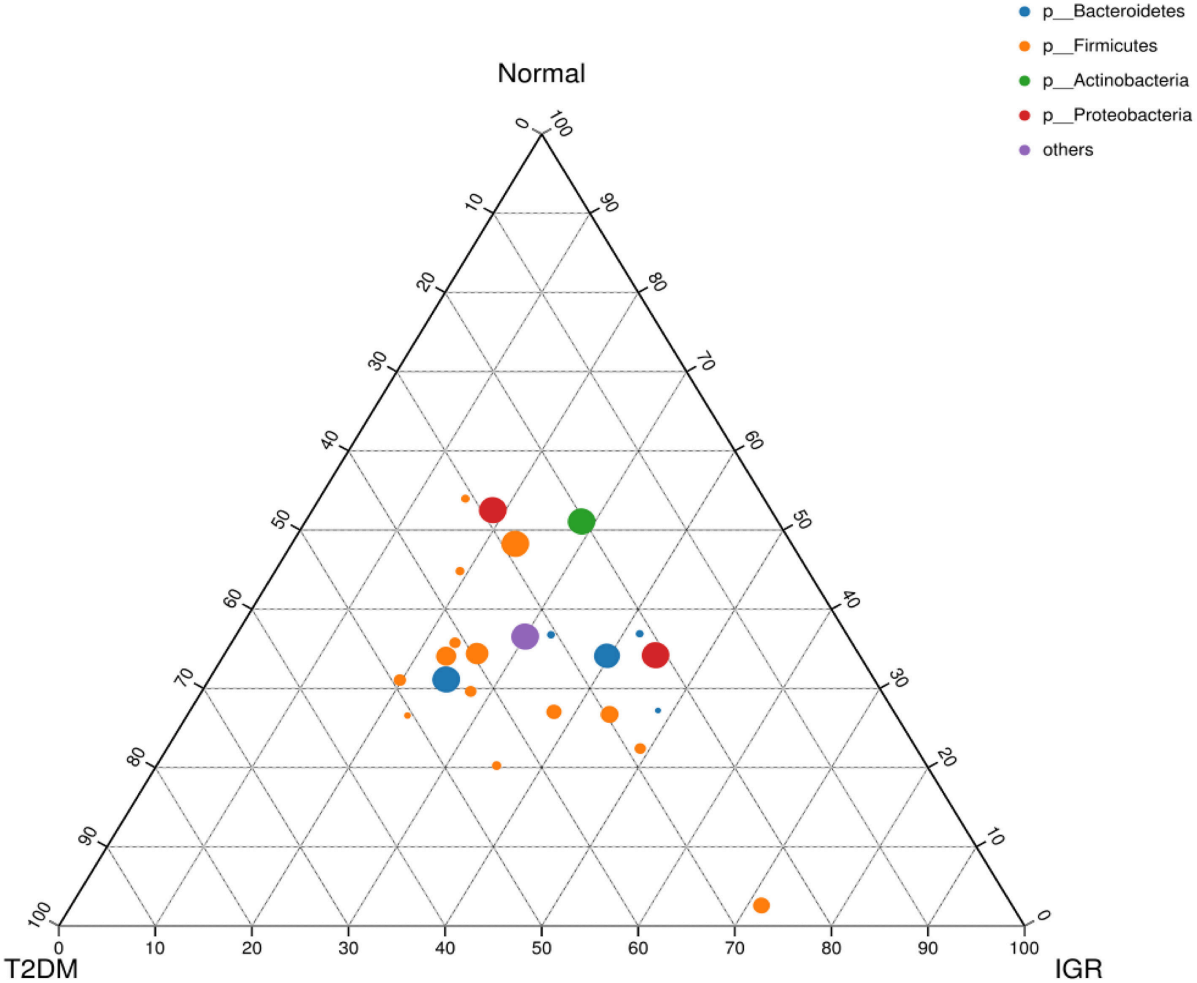
Ternary chart of three groups. Three angles represent three groups, colored circle in the triangle represents species at phylum level, and smaller circle represent species at genus level, the size of the circle represent the relative abundance of the species.

Fifteen phyla, 223 genera and 452 species were identified. At the phylum level, dominant bacterial phyla in three groups were *Firmicutes, Bacteroidetes, Proteobacteria, Actinobacteria, Verrucomicrobia, Cyanobacteria, Fusobacteria, Elusimicrobia*. Among them, *Firmicutes*, and *Bacteroidetes* had the highest abundance in three groups, *Bacteroidetes* accounted for 32.13, 38.35, and 36.41%, respectively, and *Firmicutes* accounted for 62.05, 55.53, and 52.95%, respectively. The abundance of the bacterial phyla including *Proteobacteria* and *Actinomycetes* was 2.94 and 2.13% in the T2DM group, 3.14 and 2.47% in the IGR group, and 4.79 and 4.57% in the normal control group. At the phylum classification level, the structural components of the gut microbiota of the newly T2DM, IGR, and NC showed differences. Compared with the NC, the abundance of gut microbiota in T2DM and IGR was decreased, gut microbiota diversity was reduced, the relative abundance of *Firmicutes* and *Actinobacteria* were increased, and the relative abundance of *Bacteroidetes* and *Proteobacteria* were reduced (Figures 3, 4).

**Figure 5:**
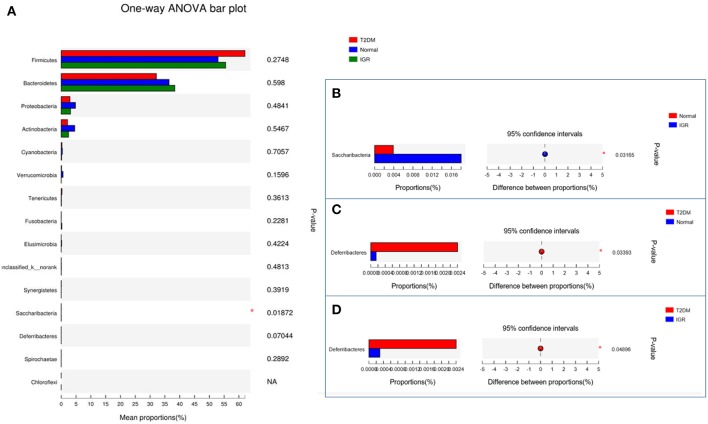
Bar plot of gut microbiota comparison at phylum level of three groups. **(A)** Gut microbiota comparison in three groups. **(B)** Saccharibacteria comparison in NC and IGR groups. **(C)** Deferribacteres comparison in NC and T2DM groups. **(D)** Deferribacteres comparison in T2DM and IGR groups; ^*^Compare groups, *P* < 0.05.

Compared to NC, *Saccharibacteria* was significantly increased in T2DM and IGR. *Deferribacteres* was significantly increased in T2DM compared to NC and IGR (Figure 5). Overall IGR patients had increased level of *Megamonas, Haemophilus, norank_p_Saccharibacteria*, had decreased levels of *Ruminococcaceae, Barnesiella, Sutterella, Ruminiclostridium, Clostridiales, Coriobacteriaceae, Flavonifractor* compared to NC, had decreased levels of *Moryella, Lachnospiraceae_NC2004_group* compared to T2DM. T2DM had increased level of *Lachnospiraceae_ND3007_group, Tyzzerella_3, norank_p_Saccharibacteria, Cetobacterium, Mucispirillum, Proteiniphilum*, had decreased level of *Barnesiella, Ruminiclostridium_9, unclassified_o_Bacteroidales* that compared to NC.

About 50% of the total microbial abundance in three groups was represented by seven genera: *Prevotella, Faecalibacterium, Bacteroides, Eubacterium_rectale_group, Megasphaera, Megamonas*, and *Dialister*.

### Linear Discriminant Effect Size (LEfSe) Analysis

**Figure 6:**
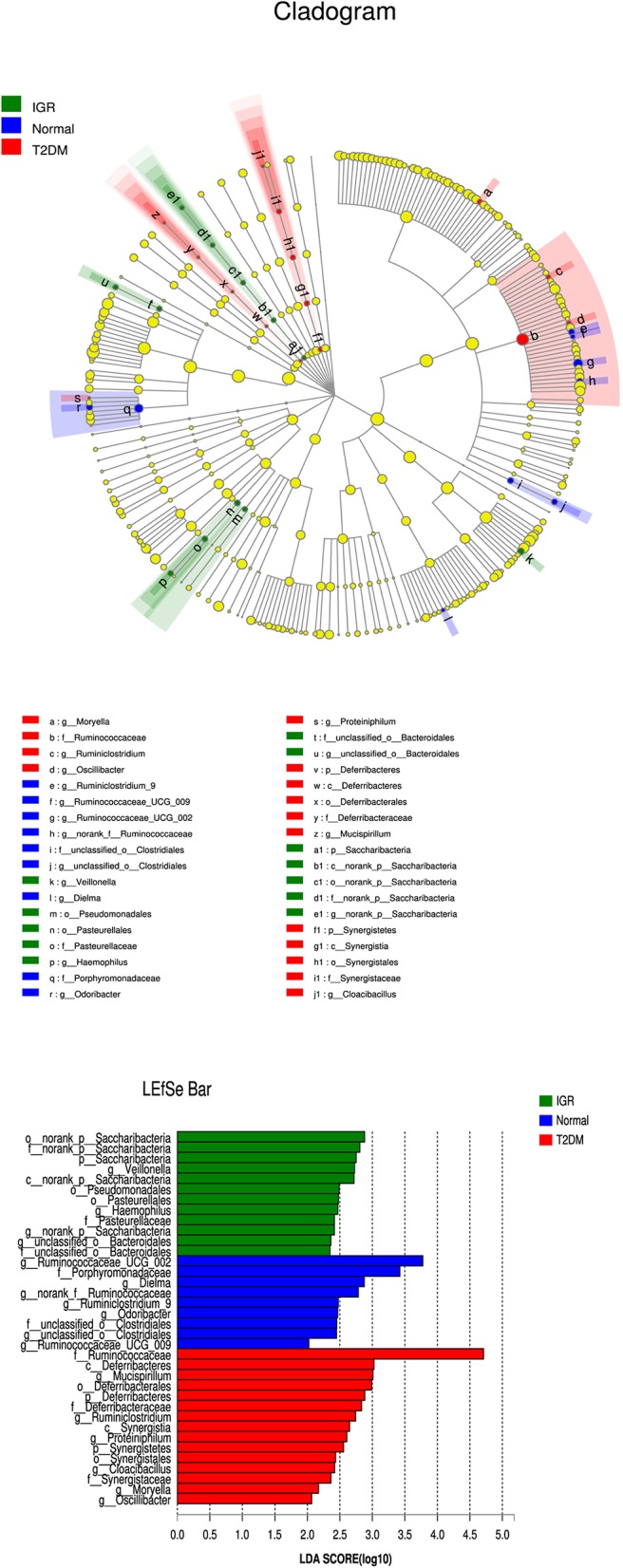
LEfSe cladogram and LDA bar chart. Circular cladogram for niche specialization of microbial compositions in three groups using the linear discriminant analysis effect size (LEfSe) analysis of the abundance patterns of bacterial taxa. The circles used in this diagram represent the taxonomic categories of organisms from the genus level as the outermost circle to phylum level as the innermost cycle. Within each given taxon, each small cycle represents its lower clade. The yellow nodes indicate no statistically significant differences of a given taxon between the samples of three groups, the red nodes indicate significantly higher relative abundance in T2DM than other two groups, the green nodes indicate significantly higher relative abundance in the IGR than other two groups, and the blue nodes indicate significantly higher relative abundance in NC than other two groups. The size of the node is in proportion to the LDA score. The links (lines) between the nodes mean hypothetically phylogenetic relationships among organisms, which can be traced back to where the lines branch off (hypothetical ancestor).

Many microbial taxa significantly differed between the T2DM, IGR, and NC groups with LDA score >2 using LEfSe analysis. We found that *Saccharibacteria, Veillonella, Pasteurellaceae*, and *Haemophilus* taxa were over-represented in IGR group; Family *Ruminococcaceae* of the phylum *Firmicutes*, genus *Mucispirillum*, and class *Deferribacteres* were apparently more abundant in the T2DM groups. Genus *Ruminococcaceae_UCG_002*, genus *Dielma*, family *Porphyromonadaceae*, genus *norank_f__Ruminococcaceae* were more abundant in NC group (Figure 6).

### Association Between Microbiota Composition and Dietary Factors

Environmental factor analysis assesses the correlation between microbes and environmental variables. There are many environmental factors related to the composition of the gut microbiota, but many of them are auto-correlated. Therefore, before the environmental factor analysis, the environmental factor screening can be prioritized, and the environmental factors with less interaction are retained for subsequent research. The environmental factors collected in this study that including basic clinical data and dietary intake. Variance inflation factor (VIF) analysis is a commonly used environmental factor screening method. By analyzing the index of the variance expansion factor >10, it can be considered as an auto-correlation environmental factor and can be excluded. The VIF values of iron and zinc elements after dietary environmental factor analysis were >10 and were therefore excluded. All environmental factors in this study were divided into four parts, which first part were basic data and clinical data, including age, gender, and blood glucose and blood lipids. The second part includes all kinds of main food intakes, the third part is about macro nutrient intake, and the fourth part related to vitamin and trace element intake. RDA and CCA analysis were used to detect the relationship between environmental factor and microbial composition.

### Correlation Study of Bacterial Phylum and Environmental Factors

**Figure 7:**
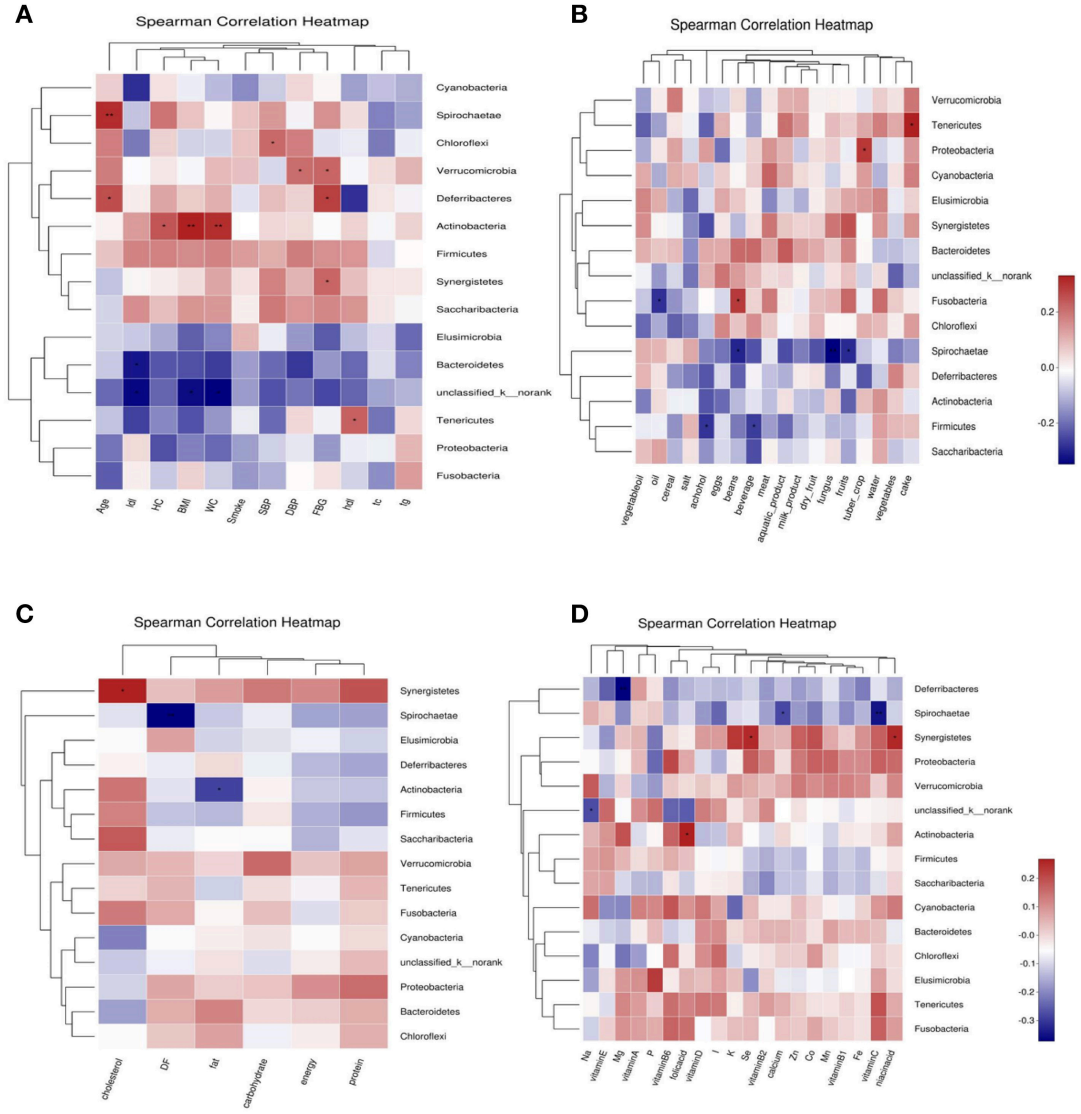
Correlation heatmap between bacterial phylum and clinical data **(A)**, Correlation heatmap between bacterial phylum and daily foods **(B)**, Correlation heatmap between bacterial phylum and daily average intake of macro nutrient **(C)**, Correlation heatmap between bacterial phylum daily average intake of Vitamins and Minerals **(D)**.

Abundance of *Bacteroidetes* was negatively correlated with LDL-C; Abundance of *Tenericutes* was positively correlated with HDL-C and cake intake; *Actinobacteria* was positively correlated with BMI, WC, HC and folic acid intake, negatively correlated with fat intake. *Firmicutes* was negatively correlated with beverage and alcohol intake. *Spirochaetae* was negatively correlated with age, and negatively correlated with fungus, fruits, beans, vitamin C, dietary fiber, and calcium intake. *Fusobacteria* was positively correlated with beans intake, and was negatively correlated with oil intake. *Proteobacteria* was positively correlated with tuber crops intake. *Synergistetes* was positively correlated with FBG, cholesterol, nicotinic acid, and selenium intake. *Deferribacteres* was negatively correlated with FBG and magnesium intake (Figure 7).

### Correlation Study of Bacterial Genus and Environmental Factors

Among the basic clinical indicators, age (*P* < 0.05), BMI, LDL-C, and HC had a greater impact on the structure of bacteria genus in the three groups; there was a positive correlation between environmental factors; *Prevotella_9* was negatively correlated with WC, HC, and BMI; *Megamonus* was positively correlated with HDL-C. *Ruminococcaceae_UGC-002* was negatively correlated with BMI; *Phascolarctobacterium* was negatively correlated SBP, BMI, WC, and HC; *Bifidobacterium* was positively correlated with BMI, WC, and HC; *Dialister* was positively correlated with SBP, DBP, and LDL-C; *Ruminococcus_1* and *Faecalibacterium* was positively correlated with age. In the analysis of food intake and bacterial genus correlation, the intake of cakes, eggs, dairy products and fruits had a great influence on the structure of bacterial genus in three groups; The intake of salt, beverage (*P* < 0.05) and alcohol (*P* < 0.05) also has a greater degree of influence on bacterial genus composition. *Prevotella_7* was positively correlated with fruits, meat and beverage intake; *Megasphaera* was positively correlated with beverage intake; *Megamonas* was positively correlated with cake intake; *Ruminococcus_1* was negatively correlated with fungus, alcohol, bean and meat intake; *Dialister* was positively correlated with water intake, negatively correlated with eggs and alcohol intake; *Subdoligranulum* was negatively correlated with crops, alcohol, and beverage intake RDA/CCA analysis of macronutrient and bacterial genus found that cholesterol intake, energy intake and protein intake had a greater impact on the bacteria composition in three groups. *Prevotella-7* was positively correlated with protein and energy intake; *Megasphaera* was positively correlated with cholesterol intake; *Dialister* was positively correlated with carbohydrate intake. The intake of vitamin A (*P* < 0.05), vitamin D, niacin and folic acid had a great influence on the structure of the bacteria; the intake of copper (*P* < 0.05), sodium and manganese also had a great influence on the structure of the bacteria; *Megasphaera* was positively correlated with Fe, Co, K and Vitamin A intake; *Dorea* was positively correlated with Mg intake; *Prevotella_7* was positively correlated with Vitamin B2, Se, Zn, and Fe intake; *Ruminococcus_2* was positively correlated with Mg and folic acid intake. *Blautia* was positively correlated with Mg (Figure S1).

## Discussion

The gut microbiota is a complex, diversified ecosystem that is symbiotic with humans. The gut microbiota and its genes and metabolites plays an important role in a series of pathophysiological processes such as pathogens invasion, establishment of immunity, nutrient digestion and absorption, body growth and metabolism, immunity and anti-tumor process, and can be combined with intestinal epithelial cells and other genes, and interact with organs and the entire human body. The diversity of intestinal microbiota is related to the occurrence of metabolic diseases such as obesity and diabetes. The interaction between gut microbiota and host may be one of the environmental risk factor for the development of T2DM (12). Diabetes is a complex clinical syndrome that arises from the interaction of environmental, genetic, and health behaviors, with environmental factors (diet, gut microbiota, age, lifestyle, and obesity); especially in T2DM that plays an important role. IGR is the pre-existing status of diabetes. Whether the regulation of gut microbiota can effectively improve the blood glucose level of IGR needs further study. Early prevention of environmental risk factors of diabetes from the IGR stage is beneficial to reduce the incidence of T2DM.

In this study, high-throughput sequencing of microbial diversity was applied to study the gut microbiota diversity between T2DM patients (*n* = 20), IGR patients (*n* = 20), and normal healthy controls (*n* = 20) with dietary survey by food frequency questionnaire (FFQ) in Uyghur population. We used strict inclusion criteria, all of Chinese Uyghur population in this study were Urumqi city citizen. T2DM patients are newly diagnosed, without using any kind of anti-diabetes medicines. Antibiotic had influence to the gut microbiota diversity (13). So we excluded people who used the antibiotics in the previous month, while excluded people who had some conditions, such as medication for hypertension, prescribed lipid lowering drugs, cardiovascular disease history, special diet, dietary supplement use, mental problems. The mean age, WC, SBP, FPG, TC of the T2DM and IGR patients were significantly (*P* < 0.01) higher than that of the NC in the dietary survey population, but we controlled the compound factors in the 60 subjects that had metagenomics study. The main regulator of the gut microbiota is including age, ethnicity, diet and the immunity. This study undergone in one ethnic population, and newly diagnosed T2DM and IGR population, reduced the interaction effect of nationality, dietary habit, and drug on the gut microbiota.

A high-fat diet may induce dysbiosis of gut microbiota, which can result in a low grade inflammatory state, obesity and other metabolic disorders (14). In our study, compared with the recommended amount of Chinese dietary guidelines and RNI, the daily intakes of vegetables, fish, shrimp and dairy products, vitamin B6, vitamin D, folic acid, calcium, and iodine were insufficient for T2DM patients, and the intake of cereals, meat, salt, oil, fat, nicotinic acid, vitamin E, potassium, iron, copper, and manganese were excessive. The daily intake of vegetables, tuber crop, fruits, nuts, cakes, cholesterol, vitamin B6, vitamin E, folic acid, calcium, phosphorus, and sodium was statistically different in three groups (*P* < 0.05). The fruit and dairy pattern may a protective factor for metabolic syndrome and hypertension and the meat eating patterns may a risk factor for fasting hyperglycemia and obesity and the Uyghur specific pattern of diet may a protective factor for fasting hyperglycemia (15). Results in our study are not completely consistent with the results of above research, which may be related to different regions where the study population was located.

The sequencing depth was adequate for all samples, and the sequencing coverage depth was >97%. The α-diversity analysis showed that the Sobs index, Shannon index, and Simpson index of the three groups were statistically significant, indicating that there were differences in gut microbiota diversity in the three groups. The Shannon index and Sobs index of the NC group larger than that of IGR group indicated that the bacterial abundance of the NC group was high; the Simpson index of the IGR group was larger than that of the NC group, indicating that the bacterial community diversity of the IGR group was low.

The bacterial structure analysis showed that the common dominant bacteria in the intestinal tract of the three groups were *Firmicutes, Bacteroidetes, Proteobacteria, Actinobacteria, Verrucomicrobia, Cyanobacteria, Fusobacteria, Elusimicrobia*, and others. *Firmicutes* is the most abundant phylum in this population, which has commonly been found to be the most abundant bacteria (16–18). *Firmicutes* and *Bacteroidetes* are the two main bacteria phylum involved in the metabolism of the host and fat accumulation. Changes in the ratio of *Bacteroidetes* to *Firmicutes* are associated with multiple disease states.

On the phylum level, *Saccharibacteria* has different abundance between three groups, the abundance of *Saccharibacteria* in the T2DM and IGR group was significantly higher than that in the NC group (*P* < 0.05). *Deferribacteres* was significantly increased in T2DM compared to NC and IGR.

The *Saccharibacteria* known as TM7 (19), is a newly discovered candidate bacteria (20). In the recent study, TM7 was cultured from human oral cavity, and showed that TM7 is extremely tiny cocci (200–300 nm) whose unique lifestyle has never been observed in human-related micro-organisms (21). In 2014, a study reported the aseptic culture of oral TM7, but did not provide sequence and culture methods (22). The TM7 in the environment is similar to TM7 in human skin and oral cavity, indicating that TM7, which is metabolically active in environmental sites, can be used as a model organism to better understand the role of TM7 in human health (23). Recently, Erin K. Crowley et al studied the effects of dietary supplements containing a mixture of magnesium-rich marine minerals on the diversity of the gastrointestinal microbiota, and found that the abundance of TM7 in the intervention group was reduced, indicating that the abundance of the bacteria has a certain correlation with trace element magnesium (24). Studies have reported that TM7 is associated with diseases such as periodontitis, vaginitis and IBD (21). In addition, this bacterium has been reported in the study of Crohn's disease and IBD, and it is shown that TM7 is associated with intestinal mucosal inflammatory diseases (25). *Saccharibacteria* may play a role in the intestinal mucosal inflammatory response of T2DM, and the mechanism needs further study.

About 50% of all bacteria were represented by seven genera: *Prevotella, Faecalibacterium, Bacteroides, Eubacterium_rectale_group, Megasphaera, Megamonas*, and *Dialister*. Overall IGR patients had increased level of *Megamonas, Haemophilus, norank_p__Saccharibacteria*, had decreased levels of *Ruminococcaceae, Barnesiella, Sutterella, Ruminiclostridium, Clostridiales, Coriobacteriaceae, Ruminiclostridium, Flavonifractor* compared to NC, had decreased levels of *Moryella, Lachnospiraceae_NC2004_group* compared to T2DM. T2DM had increased level of *Lachnospiraceae_ND3007_group, Tyzzerella_3, norank_p__Saccharibacteria, Cetobacterium, Mucispirillum, Proteiniphilum*, had decreased level of *Barnesiella, Ruminiclostridium_9, unclassified_o__Bacteroidales* that compared to NC. We found that *Saccharibacteria, Veillonella, Pasteurellaceae*, and *Haemophilus* taxa were over-represented in IGR group; Family *Ruminococcaceae* of the phylum *Firmicutes*, genus *Mucispirillum*, and class *Deferribacteres* were apparently more abundant in the T2DM groups. Genus *Ruminococcaceae_UCG_002*, genus *Dielma*, family *Porphyromonadaceae*, genus *norank_f__Ruminococcaceae* were more abundant in NC group.

*Megamonas* belongs to the *Firmicutes*, which is useful for organic nutrient, fermenting various carbohydrates, and the final products of *Megamonas* are acetic acid, propionic acid and lactic acid. Studies have shown that the abundance of *Megamonas* in the intestinal tract of Chinese population is lower than that of Africans (26), especially in the intestinal tract of centenarians (27). Chiu et al. (28) reported that the abundance of *Megamonas* in the intestines of Chinese Taiwanese obese people was higher. The abundance of *Megamonas* in the intestines of healthy people of Yao nationality in China is low, which is related to the special healthy eating habits of the ethnic group (29). Studies have shown that a decrease in *Bacteroidetes*, an increase in *Firmicutes*, or a decrease in the ratio of *Bacteroidetes* and *Firmicutes* can cause obesity, so it is proposed that the ratio of *Firmicutes* and *Bacteroidetes* can be used as a biomarker for T2DM. This study found that the abundance of *Megamonas* in the T2DM and IGR group increased, which may be related to the occurrence and development of T2DM. *Haemophilus* belongs to the *Proteobacteria*, which is a gram-negative facultative anaerobic bacterium. It is only parasitic on the intestinal mucosa of human or animal, and is sensitive to chloramphenicol, tetracycline and sulfonamide. There are several subspecies of this genus, some of which are related to clinical pathogenesis (30). This study only analyzed the differences in genus levels, and the differences in species levels are for further study. *Ruminococcaceae* belongs to the gram-positive bacteria of the thick-walled bacteria and produces butyric acid. The immune response caused by intestinal flora, especially the immune response caused by the short-chain fatty acids (SCFA) of the flora, plays an important role in the development of metabolic diseases such as diabetes (31). Intestinal bacteria can convert carbohydrates and polysaccharides in food that cannot be decomposed by the host itself into SCFA. SCFA is considered to be an important potential metabolic target in glucose metabolism and insulin resistance, preventing obesity and T2DM. The main components of SCFAs, acetic acid, propionic acid, and butyric acid, are absorbed by the intestinal mucosa, affecting 10% of the host's nutrient intake. Studies have found that people with a lack of butyrate-producing bacteria in the body are prone to T2DM, and patients with T2DM have reduced SCFAs in the intestine (32). In this study, the abundance of butyric acid producing bacteria in the IGR group was reduced, which was consistent with the results of Karlsson et al. (32).

*Barnesiella* belongs to the genus *Bacteroides* and is a newly discovered genus (33). In this study, the abundance of *Barnesiella* was reduced in the IGR group. *Sutterella* belongs to the *Proteobacteria* and is a common commensal bacteria in the human intestine. The bacteria adhere to the intestinal epithelial cells and are associated with low pre-inflammatory state of the intestine and have immunomodulatory effects (34). *Clostridiale* in *Clostridium* belongs to the *Firmicutes*, is a gram-positive anaerobic bacterium, cannot survive in an aerobic environment, and has strong spore resistance (35). Kelly et al. (36) reported that changes in the structure of *Clostridiale* are closely related to the mucosal integrity of the intestinal mucosa, and its structural changes are related to metabolic diseases such as diabetes. The abundance of *Clostridiale* in the intestinal flora of Danish pre-diabetic populations was reduced and correlated with low-grade inflammation, consistent with the results of this study (9). It is suggested that the abundance of *Clostridiale* bacteria changes during the IGR phase, and this change may play an important role in the development of diabetes.

This study found that *Bacteroidetes* was negatively correlated with LDL-C, *Tenericumes* was negatively correlated with HC and TC, *Teneriquets* was positively correlated with HDL-C, and *Actinobacteria* was positively correlated with BMI. Kasselman et al. (37) reported that the proportion of *Bacteroidetes* and *Firmicutes* in the intestinal flora of obese people was significantly lower than that of normal body mass. This study suggests that *Bacteroidetes, Firmicutes*, and *Actinobacteria* may be involved in lipid metabolism, but the mechanism is unclear. This study tried to achieve a balance between groups and reduced the impact of other confounding factors on the gut microbiota.

Diet is an important determinant of the structure and diversity of the gut microbiota. Diets that consume high fats and high sugars alter the composition of healthy microbiota, leading to microbial imbalance in the gut, a phenomenon known as “microbial dysbiosis” (38). Studies have shown that metabolic diseases such as obesity and DM are the result of interactions between gut microbiota, diet, and host (39). Numerous studies have shown that dietary intake plays an important role in shaping the gut microbiota and maintaining gut health. In healthy individuals, more than 90% of nutrients are absorbed by the small intestine and transported throughout the body to maintain the health. Complex carbohydrates (fibers) are not easily absorbed by the small intestine; enter the colon as food debris, protein residues and primary bile acids secreted by the liver in response to fat intake also enter the colon. Food entering the colon maintains intestinal health through fermentation, determines the composition and function of intestinal microbes, and plays a key role in the health of the body (40).

Dietary fiber is one of the main factors affecting microbial diversity. A comparative study of fecal samples from vegetarians, vegans, and omnivores showed that *Enterobacteriaceae, Bacteroides, Bifidobacteria* were significantly reduced in vegan compared to the omnivorous control group. *Enterobacteriaceae* levels were between vegans and controls (41). Another study conducted in remote parts of Africa and children in Europe showed that the gut microbiological structure of the two groups was significantly different, possibly related to the different dietary fiber intakes of the two groups (European children 8.4 g/day, African children 14.2 g/day) (42). Another research team conducted a two-week food exchange diet intervention experiment. After African Americans ingested a high-fiber, low-fat diet, while Africans in remote areas ingested a high-fat, low-fiber Western diet, the gut microbiota has changed in both groups (43).

Fat stimulates the secretion of bile acids, further affecting the gut microbiota. Bile acids are digested by the bacteria into secondary bile acids in the colon. Taira et al reported (44) that when the mice were changed from a low-fat diet to a high-fat diet, the structure of the gut microbiota changed, with thick-walled bacteria increasing and the number of *Bacteroidetes* decreasing. Similarly, in the another animal studies (45), when the animals were fed high-fat diet, flora changes, reducing *Lactobacillales* and *Clostridium* increase the subpopulations XIVa. However, because the mixed consumption of meat and fat is often accompanied by a decrease in fiber intake, there is still a lack of evidence that fat has a direct effect on the human gut microbiota.

The dietary fiber required for human body is usually obtained from whole grains of fruits, vegetables, and grains. Long-term dietary fiber-led diet may alter the gut microbiota, accompanied by an increase in the abundance of thick-walled bacteria, so dietary fiber may have some immunomodulatory and anti-inflammatory functions, affecting the host's immune response and function (46). In the previous discussion, it was found that an unbalanced diet leads to disorders in the structure and function of the gut microbiota, accompanied by an increase in metabolites that promote inflammation, promote proliferation, and increase disease risk. Based on evidence from epidemiological, animal, and human experimental studies, supportive diet plays an important role in the development and evolution of T2DM. For example, fiber and milk are associated with reduced risk of T2DM (47), while red meat and processed meat are associated with elevated T2DM risk, and this study has strong and consistent evidence in prospective studies (48). At the same time, dietary interventions can reshape the gut flora and alter the dietary residue entering the colon. Therefore, dietary intervention and intestinal flora intervention are promising options for preventing T2DM. Based on available evidence, it is recommended that people at high risk of T2DM adopt a balanced diet with a fiber-rich diet. Based on the results of studies on the intestinal flora and mechanisms of humans and animals, we believe that it is full of possibilities to prevent T2DM by continuing to study dietary strategies.

This study tried to achieve a balance between groups and reduced the impact of other confounding factors on the intestinal flora. However, there are many factors affecting the intestinal flora (dietary habits, lifestyle, disease status, etc.), so in order to further verify the results, it is necessary to increase the sample size for further research. This study analyzed the distribution characteristics of IGR from the perspective of intestinal flora, and tried to find the key bacteria to promote the development of diabetes, and provide theoretical basis and new therapeutic approaches for further intervention and treatment of diabetes.

## Conclusion

In summary, imbalance of intestinal microbiota may be related to the occurrence of IGR and T2DM in Uyghur population, but its mechanism needs further study. This study analyzed the distribution of intestinal microbiota IGR and newly diagnosed T2DM patients, which showed a significant decrease in the diversity and abundance of the IGR group than healthy people, and speculated that *Bacteroidetes* and *Saccharibacteria* may be related to the occurrence of T2DM. It provides a theoretical basis and a new therapeutic approach for further intervention and treatment of diabetes.

## Author Contributions

PM and RN designed and supervised this investigation. RN and JC performed this investigation. AK and YZ contributed to the data collection. All authors read and approved the final manuscript.

### Conflict of Interest Statement

The authors declare that the research was conducted in the absence of any commercial or financial relationships that could be construed as a potential conflict of interest.

## Supplementary Material

Table S1 Comparison of daily intake of foods and nutrients of three groups.

Figure S1 **(A)** Correlation heatmap between bacterial genus and clinical data. **(B)** Correlation heatmap between bacterial genus and daily average intake of foods. **(C)** Correlation heatmap between bacterial genus and macro nutrition intakes. **(D)** Correlation heatmap between bacterial genus and daily average intake of Vitamins and Minerals. Note: The different colors or shape points in the figure represent samples, the color and shape of the points represent different groupings, the distance between the points represents the similarity and difference of the samples; the green arrow represents the species; the red arrow represents the quantitative environmental factor, the environmental factor arrow The length of the environmental factor can represent the degree of influence (interpretation) of the environmental factors on the species data; the angle between the environmental factor arrows represents positive and negative correlations (sharp angle: positive correlation; obtuse angle: negative correlation; right angle: no correlation); Projecting from the sample point to the arrow of the quantitative environmental factor, the distance from the origin of the projection point represents the relative influence of the environmental factor on the sample community distribution.
