# Supplementary material for: Integrative Analysis Toward Different Glucose Tolerance-Related Gut Microbiota and Diet
Source: Front Endocrinol (Lausanne). 2019 May 27;10:295. doi: 10.3389/fendo.2019.00295 (PMC6546033; doi:10.3389/fendo.2019.00295)
Supplement: Table S1 — Comparison of daily intake of foods and nutrients of three groups. [file Table_1.DOCX]

**Table S1 |** Comparison of daily intake of foods and nutrients of three groups.

| **Foods/**  **Nutrient** | **T2DM** | **IGR** | **NC** | ***F*** | ***P*** |
| --- | --- | --- | --- | --- | --- |
| Cereals | 477.89±196.93 | 450.40±198.66 | 432.08±211.34 | 2.397 | 0.092 |
| Tuber crops | 64.54±48.08 | 92.17±76.12 | 76.75±56.14 | 7.562 | 0.001** |
| Beans | 21.35±26.69 | 24.38±17.53 | 25.35±19.21 | 1.749 | 0.175 |
| Fungus | 21.34±30.85 | 21.66±24.34 | 21.85±36.06 | 0.012 | 0.988 |
| Vegetables | 279.09±124.25 | 332.30±182.48 | 330.30±117.05 | 7.642 | 0.001^**^ |
| Fruits | 354.50±249.03 | 447.80±308.92 | 437.35±268.83 | 5.386 | 0.005* |
| Dairy products | 209.55±206.54 | 216.91±191.42 | 220.09±176.86 | 0.149 | 0.861 |
| Egg | 43.13±52.25 | 41.93±68.47 | 35.92±31.88 | 1.325 | 0.267 |
| Meats | 151.55±115.11 | 140.78±117.05 | 137.24±111.29 | 0.762 | 0.467 |
| Fish & shrimp | 10.23±19.29 | 11.69±24.32 | 7.84±14.44 | 2.175 | 0.115 |
| Cake | 12.19±22.40 | 19.55±40.22 | 27.64±81.99 | 3.048 | 0.048* |
| Nuts | 67.26±57.92 | 71.66±56.60 | 56.62±46.98 | 4.418 | 0.012* |
| Water | 1481.80±1080.99 | 1591.31±1340.90 | 1426.26±1638.50 | 0.605 | 0.546 |
| Beverage | 147.83±294.44 | 218.95±329.27 | 225.26±325.56 | 3.021 | 0.05 |
| Alcohol | 2.15±13.29 | 2.54±10.22 | 4.58±37.27 | 0.47 | 0.625 |
| Salt | 6.75±2.69 | 6.40±3.18 | 6.38±2.55 | 0.949 | 0.388 |
| Oil | 38.67±17.73 | 34.41±16.80 | 35.87±16.33 | 2.257 | 0.106 |
| Energy (kcal) | 2570.65±500.31 | 2585.92±515.05 | 2509.18±527.09 | 1.275 | 0.28 |
| Protein (g) | 96.87±26.83 | 96.38±26.92 | 93.65±28.34 | 0.831 | 0.436 |
| Fat (g) | 100.89±27.50 | 97.62±29.59 | 95.08±28.20 | 2.019 | 0.134 |
| Carbohydrate (g) | 338.94±97.72 | 352.11±92.63 | 329.80±92.89 | 2.611 | 0.074 |
| Dietary fiber (mg) | 23.02±9.84 | 25.58±11.45 | 24.59±10.36 | 2.178 | 0.114 |
| Cholesterol (g) | 379.82±172.01 | 281.52±168.40 | 248.11±142.33 | 33.82 | <0.001^**^ |
| Vitamin A  (μgRE) | 1053.08±423.45 | 1080.71±361.40 | 1089.89±266.60 | 0.574 | 0.564 |
| Vitamin B1  (mg) | 1.48±0.56 | 1.49±0.57 | 1.47±0.61 | 0.099 | 0.906 |
| Vitamin B2  (mg) | 1.50±0.58 | 1.56±0.60 | 1.61±0.69 | 1.345 | 0.261 |
| Vitamin B6  (mg) | 0.47±0.18 | 0.57±0.25 | 0.54±0.24 | 6.538 | 0.002^*^ |
| Vitamin C (mg) | 131.10±53.30 | 127.25±51.03 | 154.29±81.69 | 10.763 | <0.001^**^ |
| Vitamin D (μg) | 1.51±1.37 | 1.52±1.69 | 1.29±1.06 | 2.052 | 0.129 |
| Vitamin E (mg) | 35.20±12.13 | 43.15±12.81 | 48.89±13.54 | 53.111 | <0.001^**^ |
| Folic acid (μg) | 113.16±55.70 | 127.25±51.03 | 130.60±53.93 | 5.132 | 0.006^*^ |
| Nicotinic acid (mg) | 22.40±10.91 | 21.52±9.66 | 20.57±8.83 | 1.79 | 0.168 |
| Calcium (mg) | 642.93±254.29 | 735.46±304.85 | 728.72±280.45 | 5.33 | 0.005^*^ |
| Phosphorus  (mg) | 971.82±315.00 | 1021.54±320.38 | 1089.03±271.20 | 7.94 | 0.001^**^ |
| Potassium (mg) | 2515.91±541.81 | 2610.20±579.97 | 2518.20±510.71 | 1.56 | 0.212 |
| Sodium (mg) | 3026.47±537.11 | 2835.13±729.26 | 2882.81±633.59 | 3.64 | 0.027* |
| Magnesium  (mg) | 410.51±92.20 | 424.03±90.35 | 413.17±95.36 | 0.87 | 0.421 |
| Iron (mg) | 28.18±9.07 | 28.97±9.43 | 27.95±9.80 | 0.54 | 0.581 |
| Iodine (μg) | 32.49±18.38 | 35.43±24.38 | 34.17±21.52 | 0.67 | 0.512 |
| Zinc (mg) | 17.22±6.67 | 17.46±7.47 | 16.82±7.11 | 0.42 | 0.658 |
| Selenium  (μg) | 78.95±27.50 | 75.37±25.84 | 74.27±27.32 | 1.45 | 0.236 |
| Copper (mg) | 4.00±1.87 | 4.30±2.04 | 4.27±2.04 | 1.08 | 0.339 |
| Manganese  (mg) | 7.14±2.30 | 7.25±2.27 | 7.00±2.60 | 0.47 | 0.626 |

Notes: * (*P*<0.05), ** (*P*<0.01)
