# Supplementary figures and images for: Integrative Analysis Toward Different Glucose Tolerance-Related Gut Microbiota and Diet
Source: Front Endocrinol (Lausanne). 2019 May 27;10:295. doi: 10.3389/fendo.2019.00295 (PMC6546033; doi:10.3389/fendo.2019.00295)

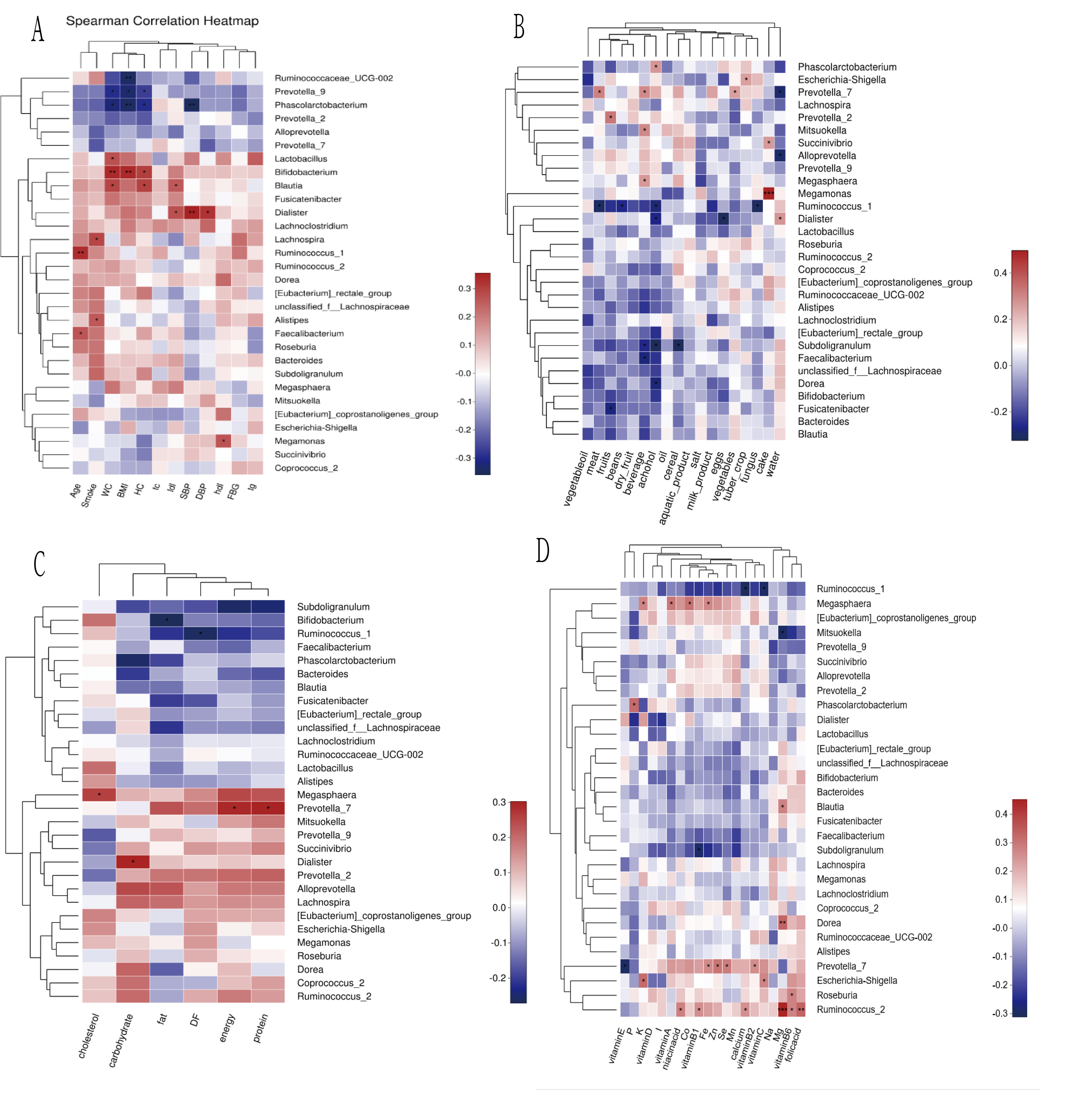

Supplement: Figure S1 — (A) Correlation heatmap between bacterial genus and clinical data. (B) Correlation heatmap between bacterial genus and daily average intake of foods. (C) Correlation heatmap between bacterial genus and macro nutrition intakes. (D) Correlation heatmap between bacterial genus and daily average intake of Vitamins and Minerals. Note: The different colors or shape points in the figure represent samples, the color and shape of the points represent different groupings, the distance between the points represents the similarity and difference of the samples; the green arrow represents the species; the red arrow represents the quantitative environmental factor, the environmental factor arrow The length of the environmental factor can represent the degree of influence (interpretation) of the environmental factors on the species data; the angle between the environmental factor arrows represents positive and negative correlations (sharp angle: positive correlation; obtuse angle: negative correlation; right angle: no correlation); Projecting from the sample point to the arrow of the quantitative environmental factor, the distance from the origin of the projection point represents the relative influence of the environmental factor on the sample community distribution. [file Image_1.TIF]
